# Targeted next generation sequencing in 112 Chinese patients with intellectual disability/developmental delay: novel mutations and candidate gene

**DOI:** 10.1186/s12881-019-0794-y

**Published:** 2019-05-14

**Authors:** Huifang Yan, Zhen Shi, Ye Wu, Jiangxi Xiao, Qiang Gu, Yanling Yang, Ming Li, Kai Gao, Yinyin Chen, Xiaoping Yang, Haoran Ji, Binbin Cao, Ruoyu Duan, Yuwu Jiang, Jingmin Wang

**Affiliations:** 1 Department of Pediatrics, Peking University First Hospital, Beijing, China; 2 Beijing Key Laboratory of Molecular Diagnosis and Study on Pediatric Genetic Diseases, Peking University First Hospital, Beijing, China; 3 Department of Radiology, Peking University First Hospital, Beijing, China; 4 Department of Neurology, First Hospital of Shanxi Medical University, Taiyuan, China; 5 VIP Ward, Affiliated Hospital of Hebei University, Baoding, China; 6 Children’s Hospital of Zhejiang University School of Medicine, Hangzhou, China; 7 Guangzhou Women and Children’s Medical Center, Guangzhou, China; 8 Key Laboratory for Neuroscience, Ministry of education/National Health and Family Planning Commission, Peking University, Beijing, China

**Keywords:** Developmental delay, Intellectual disability, Genetic diagnosis, Next generation sequencing, Pathogenicity

## Abstract

**Background:**

Intellectual disability/developmental delay is a complex condition with extraordinary heterogeneity. A large proportion of patients lacks a specific diagnosis. Next generation sequencing, enabling identification of genetic variations in multiple genes, has become an efficient strategy for genetic analysis in intellectual disability/developmental delay.

**Methods:**

Clinical data of 112 Chinese families with unexplained intellectual disability/developmental delay was collected. Targeted next generation sequencing of 454 genes related to intellectual disability/developmental delay was performed for all 112 index patients. Patients with promising variants and their other family members underwent Sanger sequencing to validate the authenticity and segregation of the variants.

**Results:**

Fourteen promising variants in genes *EFNB1, MECP2, ATRX, NAA10, ANKRD11, DHCR7, LAMA1, NFIX, UBE3A, ARID1B* and *PTPRD* were identified in 11 of 112 patients (11/112, 9.82%). Of 14 variants, eight arose de novo*,* and 13 are novel. Nine patients (9/112, 8.03%) got definite molecular diagnoses. It is the first time to report variants in *EFNB1, NAA10, DHCR7, LAMA1* and *NFIX* in Chinese intellectual disability/developmental delay patients and first report about variants in *NAA10* and *LAMA1* in affected individuals of Asian ancestry.

**Conclusions:**

Targeted next generation sequencing of 454 genes is an effective test strategy for patients with unexplained intellectual disability/developmental delay. Genetic heterogenicity is significant in this Chinese cohort and de novo variants play an important role in the diagnosis. Findings of this study further delineate the corresponding phenotypes, expand the mutation spectrum and support the involvement of *PTPRD* in the disease.

**Electronic supplementary material:**

The online version of this article (10.1186/s12881-019-0794-y) contains supplementary material, which is available to authorized users.

## Background

Intellectual disability/developmental delay (ID/DD) is a common group of neurodevelopmental disorders with a prevalence of 1% ~ 3%, which starts before the age of 18 years and is characterized by substantial limitations in intellectual functioning and adaptive behavior [1]. ID cannot be diagnosed until the child is older than five years old, when standardized measures of developmental skills, such as the Wechsler Intelligence Scale for Children (WISC), are more reliable and valid. DD is defined as delay in two or more developmental domains, including gross or fine motor, speech/language, social/personal, cognitive and activities of daily living. DD can be assessed by Gesell Developmental Scale and mostly predicts a future diagnosis of ID. Although ID/DD can also be caused by exogenous factors such as maternal alcohol abuse during pregnancy, birth complications, infections and extreme malnutrition, genetics plays a vital role in its etiology [2]. Discerning the precise genetic causes of ID/DD patients will inform prognosis, management and therapy, enable access to disorder-specific support groups, and facilitate family planning [3]. Unfortunately, due to extreme genetic heterogeneity, genetic causes are remaining to be clarified in most ID/DD patients [4].

Genomic variants including structural variants and sequence variants can both lead to ID/DD. The former encompasses both copy number variants (CNVs) and balanced rearrangements and can be detected by conventional karyotyping and chromosomal microarray analysis (CMA), explaining up to 15% of ID/DD cases [1, 2]. Sequence variants may cause monogenic disorders and can be discovered by DNA sequencing. In recent years, next generation sequencing (NGS), enabling identification of genetic variations in multiple genes, has become an effective strategy for genetic analysis in ID/DD. Based on NGS technology, three diagnostic tests including targeted NGS, also known as gene panel, whole exome sequencing (WES) and whole genome sequencing (WGS) sequencing are currently used for diagnosis of ID/DD. The main differences among the three tests are the different range of the targeted sequenced regions. Targeted NGS focuses on hundreds of disorder-specific genes. By contrast, WES covering all ~ 20,000 protein-coding genes and WGS sequencing all the entire genomes are non-targeted tests [5]. With more genome regions covered, WES and WGS get a higher diagnostic rate of ID/DD (~ 40% and ~ 42%, respectively) compared with targeted NGS (11%~ 32%) [3, 6–9]. However, given its lower cost, deeper coverage depth, easier data management, targeted NGS is still a common approach in routine clinical diagnostic laboratories. In this study, targeted NGS for 454 genes related to ID/DD was performed for 112 Chinese patients with unexplained ID/DD to elucidate their genetic causes and enable access to further medical management.

## Methods

### Patients

Patients with unexplained ID/DD were defined as those who did not get an etiological diagnosis after prior etiology tests including screening for inborn errors of metabolism and karyotype analysis. The inclusion criteria were: 1) age at first exam was from 3 months to 18 years; 2) ID: IQ < 70, assessed by WISC; DD: DQ < 76 in two or more developmental domains assessed by Gesell Developmental Scale; 3) no history of perinatal brain injury, postnatal hypoxia, intoxication, cranial trauma or central nervous system infection; 4) no evidence of recognizable inherited metabolic disorder or neurodegenerative disorders.

All 112 Chinese patients were examined and enrolled by pediatric neurologists in Peking University First Hospital from May of 2014 to August 2016. Genomic DNA of each index patient and his or her parents or other family members were extracted from peripheral leukocyte using Flexi Gene DNA Kit (QIAGEN, Germany) according to standard procedure.

### Targeted NGS

A panel of 454 ID/DD-related genes (Additional file 1) was developed. Targeted NGS including exons capture and sequencing on an Illumina GAIIx platform (Illumina, San Diego, CA, U.S.A.) was performed for each index patient by Kangso Medical Inspection (Beijing). Variants were annotated by Variant Effect Predictor [10] and filtered according to the following criteria: 1) nonsynonymous SNV or indel located in exonic or splicing regions; 2) absent from controls or allele frequency < 0.01 and no homozygotes or hemizygotes if recessive in Exome Sequencing Project (ESP) [11], 1000 Genomes Project (1000G) [12], Exome Aggregation Consortium (ExAC) or Genome Aggregation Database (gnomAD) [13]; 3) predicted to be damaging by at least four of the following six software: SIFT [14], Polyphen2 [15], Mutationtaster [16], CADD [17], M-CAP [18], Condel [19] and PROVEAN [20]. For splice variants, Human Splicing Finder [21] and Splice Site Prediction [22] were used to estimate the impact of the splice site change on the transcripts. Variants were classified as “pathogenic,” “likely pathogenic,” “uncertain significance,” “likely benign,” and “benign” according to ACMG guideline and relative annotation files [23–25].

Sanger sequence was performed for index patients and other family members to validate the authenticity and segregation of the promising variants.

### Statistical analyses

Differences were analyzed statistically using the Chi-square test by IBM SPSS Statistics 19.

## Results

Of 112 Chinese patients, 69 were males, and 43 were females. The median age was 3 years and 7 months [range 4 months–17 years]. 49 patients, older than 5 years of age, were diagnosed with ID, while the remaining 63 patients, younger than 5 years of age, were diagnosed with DD. 18 patients (18/112, 16.07%) presented with mild ID/DD, while the remaining 94 patients (94/112, 83.93%) were affected by moderate to severe ID/DD. Congenital malformation, abnormal behavior, epilepsy, positive family history and MRI abnormality were observed in 52.68, 22.32,17.86, 13.39 and 59.70% of patients, respectively. There was no statistical difference (*p* >0.05) in the ratio of gender (male versus female) and the severity of ID/DD (mild delay versus moderate and severe delay), and the incidence of the congenital malformation, abnormal behavior, epilepsy, positive family history and MRI abnormality between two groups of patients with or without meaningful targeted NGS results (Table 1).

**Table 1:** Clinical characteristics of 112 ID/DD patients

Group	N	Gender (M/F)	ID/DD (mil/mod~)	Malfor- mation (n, %)	Abnormal behavior (n, %)	Epilepsy (n, %)	Family history (n, %)	MRI abnormality (n/N, %)
Total	112	69/43	18/94	59, 52.68	25, 22.32	20,17.86	15,13.39	40/67, 59.70
Positive NGS	11	6/5	3/8	7, 63.64	3, 27.27	3, 27.27	2, 18.18	4/7, 57.14
Negative NGS	101	63/38	15/86	52, 51.49	22, 21.78	17,16.83	13, 12.87	36/60, 60.00
x^2^		0.26	1.13	0.59	0.17	0.74	0.24	0.21
p		0.61	0.29	0.44	0.68	0.39	0.62	0.88

Note: *N* number, *ID/DD* intellectual disability/developmental delay, *NGS* targeted next generation sequencing**,** *M* male, *F* female, *mil* mild delay, *mod~* moderate or severe delay, Malformation included appearance malformations (dysmorphic face, single transverse palmar crease) and organ abnormality in heart or kidney

In 11 of 112 patients (11/112, 9.82%), 14 promising variants were detected in 11 genes *EFNB1, MECP2, ATRX, NAA10, ANKRD11, DHCR7, LAMA1, NFIX, UBE3A, ARID1B* and *PTPRD*. Of 14 variants, 13 were absent from ESP, 1000G, ExAC and gnomAD databases, whereas one was with a frequency of 1.85e-3 in East Asian population (gnomAD database). Variant spectrum consisted of seven missense variants, five nonsense variants, one frameshift variant and one splice variant. All variants are predicted to be damaging based on multiple prediction tools. 6 of 14 (6/14,42.86%) variants were inherited from parents, and the remaining eight (8/14, 57.14%) arose de novo. Except for the variant in *MECP2*, which was reported before [26], the remaining 13 variants are novel (Table 2).

**Table 2:** Characteristics of 14 variants identified in 11 patients with intellectual disability/developmental delay

Pt	G	Age	Gene	Inh	Reference	CDS	Protein	Or	N/R	Pat	AF	GERP	CADD	SI	Po	MT	Co	PR	MC
1	F	16Y	*EFNB1*	XL	NM_004429.4	c.640C > T	p.(Gln214Ter)	d	N	LP	–	5.2	38.0	–	–	D	–	–	–
2	F	1Y3M	*MECP2*	XL	NM_004992.3	c.808C > T	p.(Arg270Ter)	d	R	P	–	3.8	36.0	–	–	D	–	–	–
3	M	17Y	*ATRX*	XL	NM_000489.3	c.6257 T > C	p.(Leu2086Ser)	m	N	LP	–	5.5	31.0	–	D	D	–	D	D
4	M	1Y7M	*ATRX*	XL	NM_000489.3	c.6679G > T	p.(Asp2227Tyr)	d	N	LP	–	5.7	33.0	–	D	D	–	D	D
5	M	1Y3M	*NAA10*	XL	NM_003491.3	c.248G > A	p.(Arg83His)	m^a^	N	LP	–	5.0	34.0	D	D	D	D	D	D
			*ANKRD11*	AD	NM_013275.5	c.884G > A	p.(Ser295Asn)	d	N	VUS	–	5.9	31.0	D	D	D	D	N	T
6	F	8Y4M	*DHCR7*	AR	NM_001163817.1	c.1376G > A	p.(Trp459Ter)	p	N	P	–	5.1	42.0	–	–	D	–	–	–
						c.901C > A	p.(His301Asn)	m	N	LP	–	3.6	25.7	D	D	D	D	D	D
7	F	9Y4M	*LAMA1*	AR	NM_005559.3	c.1711_1712del	p.(Ala571Pro fsTer8)	p	N	VUS	–	–	–	–	–	–	–	–	–
						c.2755G > C	p.(Gly919Arg)	m	N	LP	1.85e-3	5.5	29.2	D	D	D	D	D	D
8	M	4Y2M	*NFIX*	AD	NM_001271043.1	c.613C > T	p.(Gln205Ter)	d	N	P	–	4.6	39.0	–	–	D	–	–	–
9	M	3Y3M	*UBE3A*	AD	NM_000462.3	c.403G > T	p.(Glu135Ter)	d	N	P	–	5.8	37.0	–	–	D	–	–	–
10	M	1Y	*ARID1B*	AD	NM_001346813.1	c.6212 T > A	p.(Ile2071Asn)	d	N	LP	–	5.4	32.0	D	D	D	D	D	D
11	F	5Y5M	*PTPRD*	AD?	NM_002839.3	c.5534 + 1G > A	p.(Ser1845Arg fsTer2)	d	N	–	–	−10.0	–	–	–	D	–	–	–

Note: *Pt* patient, *G* gender, *Y* years, *M* months, *Inh* inheritance pattern, *XL* X-linked, *AD* autosomal dominant, *AR* autosomal recessive, *Or* origin, *m* maternal, *p* paternal, *d* de novo, *N* novel, *R* reported, *Pat* pathogenicity, *P* pathogenic, *LP* likely pathogenic, *VUS* variant with uncertain significance, *GERP* GERP++RS score, *D* “probably damaging” in Polyphen2 or “deleterious” in other software, *SI* SIFT, *Po* PolyPhen2, *MT* MutationTaster, *Co* Condel, *PR* PROVEAN, *MC* M-CAP;^a^, the variant arose de novo in the patient’s mother

Among 11 mutated ID genes, there were four X-linked genes *EFNB1* (MIM* 300035), *MECP2* (MIM* 300005), *ATRX* (MIM* 300032) and *NAA10* (MIM* 300013), two autosomal recessive genes *DHCR7* (MIM* 602858) and *LAMA1* (MIM* 150320), four autosomal-dominant genes *ANKRD11* (MIM* 611192), *NFIX* (MIM* 164005), *UBE3A* (MIM* 601623) and *ARID1B* (MIM* 614556) and one candidate gene *PTPRD* (MIM* 601598). Except for *ATRX* reoccurring two times, the remaining 10 genes were detected in only one patient. *NAA10* and *ANKRD11* were mutated in the same patient.

### Variants in X-linked ID/DD genes

**Fig. 1:**
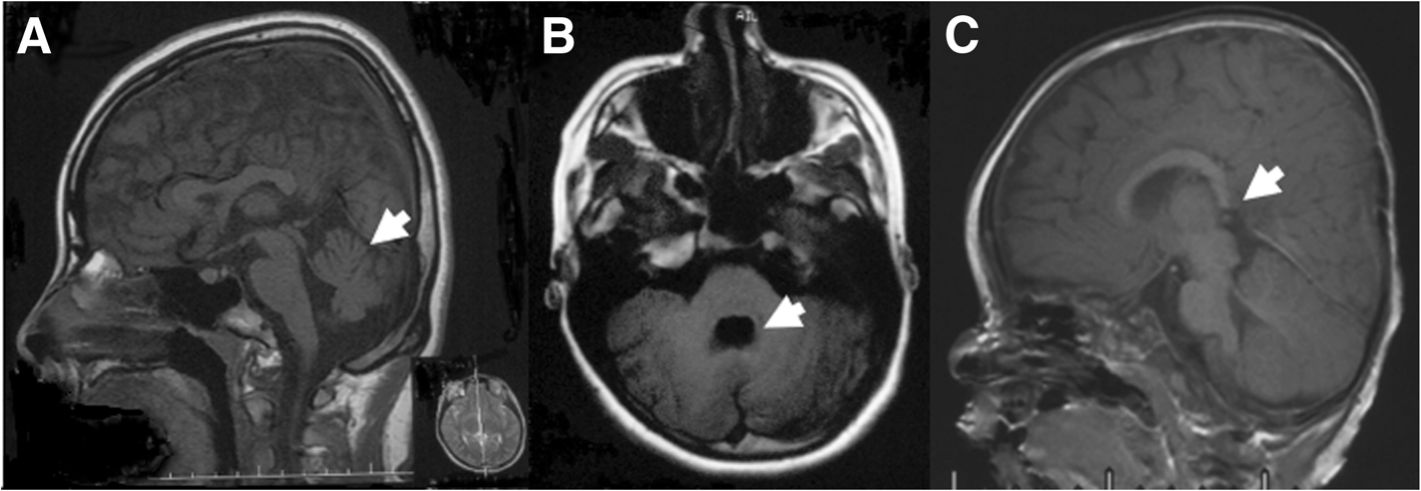
Brain abnormality of Patient 1 and Patient 10. (**a**, **b**) T1 weighted image of Patient 1 examined at the age of 15 years showed cerebellar vermis dysplasia (**a**) and fourth ventricle deformity (**b**). (**c**) T1 weighted image of Patient 10 examined at the age of 9 months showed dysplasia of the splenium of corpus callosum

De novo heterozygous variant c.640C > T; p.(Gln214Ter) in *EFNB1* (NM_004429.4) was detected in Patient 1, a girl at the age of 16 years. Her phenotype included severe ID, typical facial dysmorphia (brachycephaly, frontal bossing, widow’s peak, hypertelorism, telecanthus, bifid nasal tip and curly hair), strabismus, myopia, astigmatism, short fifth finger, cerebellar vermis dysplasia and fourth ventricle deformity (Fig. 1a, b). No malformation in mouth, nails, skin or chest were noted.

Patient 2 harbored de novo heterozygous variant c.808C > T; p.(Arg270Ter) in *MECP2* (NM_004992.3). Evaluated at the age of 1 year and 3 months, the girl’s main symptoms were DD, hand wringing and sleep disturbance. Developmental regression, microcephaly and seizures were not recorded yet.

**Fig. 2:**
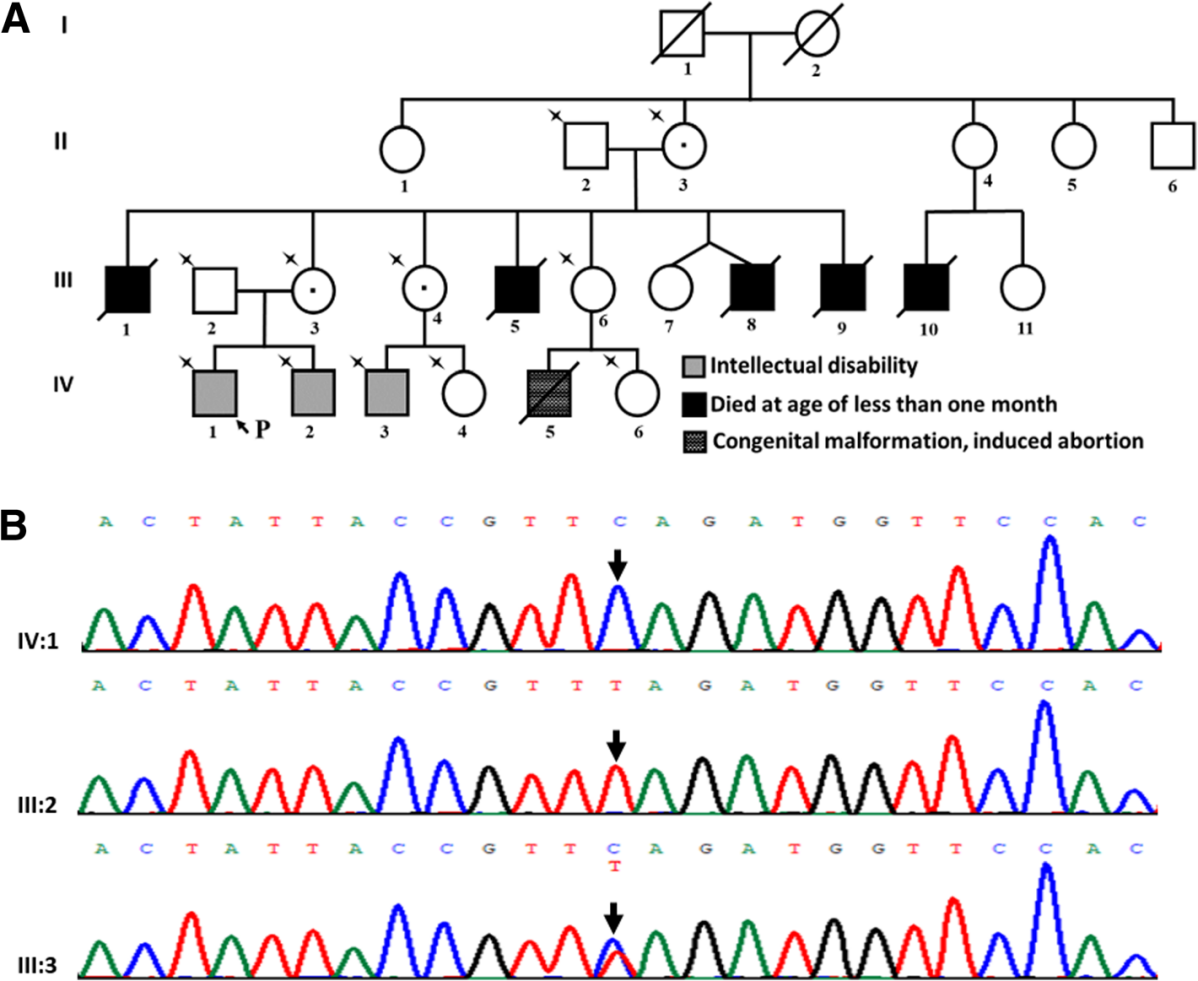
The variant in *ATRX* in Patient 3. (**a**) Pedigree of Patient 3. Arrow and P indicate the proband; star indicates members who underwent Sanger sequencing. (**b**) *ATRX* sequence result of Patient 3 and his parents. A hemizygous variant c.6257 T > C; p.(Leu2086Ser) in *ATRX* (NM_000489.3) was identified in the proband (IV:1). His mother (III:3) was in heterozygous state for the variant and his father (III:2) was wild type at the same site

A hemizygous variant c.6257 T > C; p.(Leu2086Ser) in *ATRX* (NM_000489.3) was detected in Patient 3, who was characterized by moderate ID, dysmorphic face (large forehead, low anterior hairline, hypertelorism, broad nasal bridge, small ears, strabismus), ventricular septal defect (repaired at the age of 5 years), scoliosis, and high arch of left foot. No microcephaly, genitourinary malformation, deafness or signs of anemia including hepatosplenomegaly, anemia-like bone changes, jaundice or abnormal red blood cell indices were observed. He had a complicated family history (Fig. 2). Sanger sequencing for family members (Fig. 2. II:2, II:3, III:2, III:3, III:4, III:6, IV:1, IV:2, IV:3, IV:4, IV:6) showed that his grandmother (II:3), mother (III:3), and aunt (III:4) all carried the variant. His younger brother (IV:2) and the son of his aunt (IV:3) presented with the same variant, both of whom were also affected by ID. Co-segregation of the variant with the phenotype support the pathogenicity of the variant. Notably, five uncles (III:1, III:5, III:8, III:9, III:10) of Patient 3 all died in the first months of life with unknown causes. It is unclear whether the recurrent early death is associated with the variant in *ATRX*.

Patient 4 had a different hemizygous variant c.6679G > T; p.(Asp2227Tyr) in *ATRX* (NM_000489.3), which arose de novo. The neurologic symptoms of Patient 4 included severe DD (could not speak, control his head, sit, stand, or walk at 1 year and 7 months of age), early onset epilepsy (3 months) and enlarged brain ventricle. Except for dysmorphic face, no other congenital malformations, deafness or signs of anemia were noted.

Hemizygous variant c.248G > A; p.(Arg83His) in *NAA10* (NM_003491.3) was detected in Patient 5, a boy demonstrating moderate DD, hyperactivity, electrocardiographic T-wave abnormality and delayed bone age evaluated at the age of 4.5 years. Further validation showed that the variant arose de novo in the patient’s mother, who had borderline ID. Of note, the boy also had a de novo heterozygous variant c.884G > A; p.(Ser295Asn) in *ANKRD11* (NM_013275.5).

### Variants in autosomal recessive ID/DD genes

Compound heterozygous variant c.901C > A; p.(His301Asn) and c.1376G > A; p.(Trp459Ter) in *DHCR7* (NM_001163817.1) were detected in Patient 6, a girl at the age of 8 years and 4 months. Her main clinical features were mild ID and facial dysmorphia including microcephaly, hypertelorism, narrow palpebral fissures and broad nasal bridge. No fingers/toes abnormalities were noted. The Patient did not undergo 7-dehydrocholesterol measurement to confirm the diagnosis further.

Patient 7 harbored compound heterozygous variants c.1711_1712del; p.(Ala571ProfsTer8) and c.2755G > C; p.(Gly919Arg) in *LAMA1* (NM_005559.3). Her main complains were ID, epilepsy and sinus block. No ataxia or ocular anomalies were noted evaluated at the age of 9 years and 4 months. The patient was equipped with a pacemaker and could not undergo MRI examination. Therefore, it was unknown whether Patient 7 had brain abnormality or not. To clarify if the patient had any other promising damaging variants, trio-based WES was performed for Patient 7 and her parents. Interestingly, except variants in *LAMA1*, no other promising variants stood out.

### Variants in autosomal dominant ID/DD genes

De novo heterozygous variant c.613C > T; p.(Gln205Ter) in *NFIX* (NM_001271043.1) was detected in Patient 8. The boy, at the age of 4 years and 2 months, was clinically suspected as Sotos syndrome (MIM# 614753) with facial dysmorphia (long and narrow face, high forehead and downslanting palpebral fissures), mild DD, significant delay in language (started to speak at the age of 3 years) and overgrowth. Previous fluorescence in situ hybridization (FISH) did not detect the deletion of 5q35 region, a common defect leading to Sotos syndrome.

De novo heterozygous variant c.403G > T; p.(Glu135Ter) in *UBE3A* (NM_000462.3) was identified in Patient 9. Evaluated at the age of 3 years and 3 months, the boy presented with global DD, significantly delayed language (starting to speak at 3 years of age), epilepsy (onset at 2 years of age), inappropriate smile, microcephaly and facial dysmorphia (hypertelorism, small ear, long philtrum and prominent jaw). Clinical diagnosis of Angelman syndrome (MIM# 105830) was established. Deletion of 15q11-q13 was excluded by multiplex ligation-dependent probe amplification (MLPA).

De novo missense variant c.6212 T > A; p.(Ile2071Asn) in *ARID1B* (NM_001346813.1) was identified in Patient 10. The boy, at the age of 1 year, presented with mild DD, coarse face (low anterior hairline, thick eyebrows, broad nasal tip, long philtrum, thin upper vermilion and low-set ears), nystagmus, strabismus, delayed dentition, single transverse palmar crease, prominent distal phalanges of 4th toe of right foot, delayed myelination and agenesis of splenium of corpus callosum (Fig. 1c).

### A variant in candidate gene *PTPRD*

**Fig. 3:**
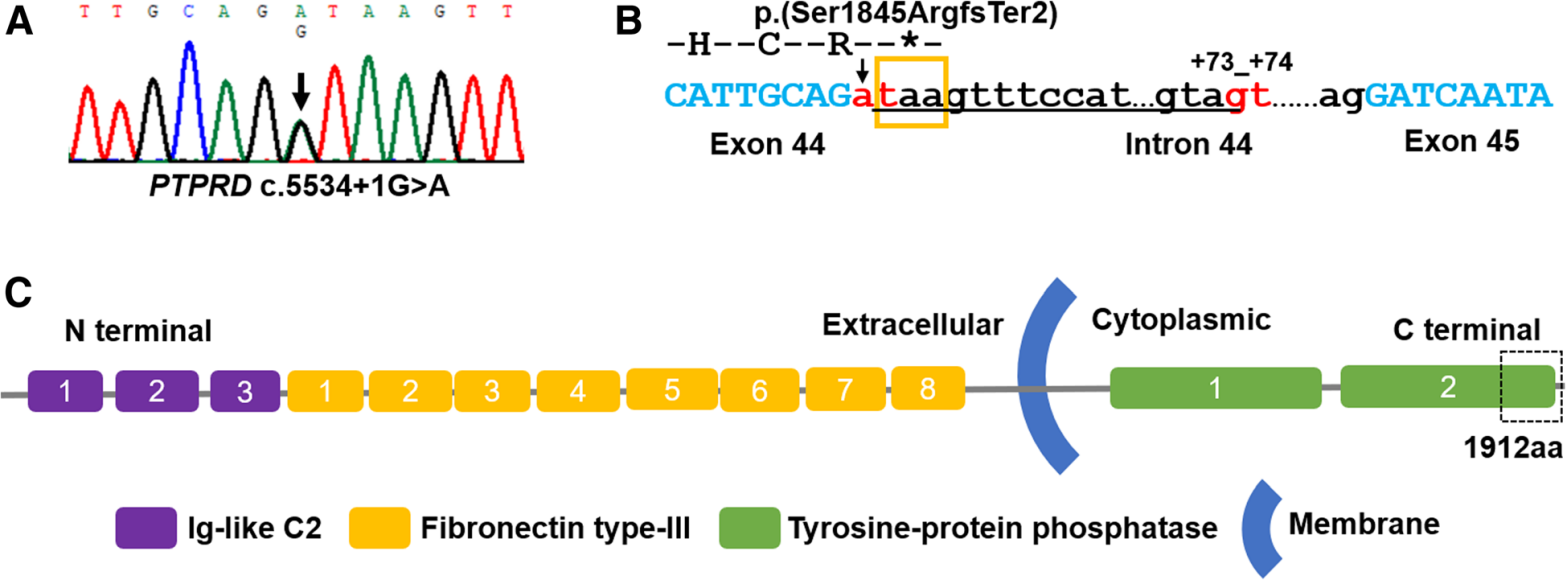
The variant in *PTPRD* in Patient 11. (**a**) *PTPRD* sequence result of Patient 11. Arrow indicates the mutation site of c.5534 + 1G > A in *PTPRD*. (**b**) Predicted change in splice donor site of intron 44. The variant c.5534 + 1G > A disrupted the wild type splice donor site (arrow and red “at”) and created a new potential splice donor site at c.5534 + 73_5534 + 74GT (red “gt”). The abnormal splicing will cause retention of 72 nucleotides on upstream of the new donor site in intron 44 (underlined), create a premature stop codon at position 1846 (orange box), and lead to premature truncation of the protein p.(Ser1845ArgfsTer2). (**c**) PTPRD protein structure. PTPRD protein is a single-pass type I membrane protein and predicted to contain conserved function domains: three Ig-like C2 domains (Ig-like C2 type1, Ig-like C2 type2 and Ig-like C2 type3) (Purple), eight fibronectin type-III domains (Orange), and two tyrosine-protein phosphatase domains (Green) (https://www.uniprot.org/uniprot/P23468). As predicted, the truncated protein (p.(Ser1845ArgfsTer2)) will lack part of tyrosine-protein phosphatase 1 domain (dotted box)

Patient 11 was a girl with moderate nonsyndromic DD. She was able to walk alone and speak at 16 months and 3.5 years, respectively. At the age of 5 years and 5 months, she attended special training school and could complete routine communication. She harbored a de novo canonical + 1 splice site variant c.5534 + 1G > A in *PTPRD* (NM_002839.3) (Fig. 3a). The variant is not seen in control population databases. Although the variant site is not conserved with a low GERP++RS score (− 10.0) [27], the variant is predicted to disrupt the wild type splice donor site in intron 44 with a consensus value (CV) of − 26.8% in HSF and a dropping SSP’s prediction score from 1 to 0. As predicted, a new splice donor site at c.5534 + 73_5534 + 74GT with a high SSP’s prediction score of 0.97 will be created. The altered splicing will cause the retention of 72 nucleotides in intron 44, change the reading frame and lead to a premature stop codon at position 1846 (p.(Ser1845ArgfsTer2)) (Fig. 3b). The changed/missing region p.1845_1912 of the predicted mutated protein is a part of tyrosine-protein phosphatase 1 domain (Fig. 3. **c**), which is highly conserved in all seven isoforms of PTPRD protein.

## Discussion

In this study, through targeted sequencing of 454 ID/DD genes, promising variants were identified in 9.82% of patients. Same as expected, genetic heterogenicity was significant in this Chinese cohort. 11 patients presented with 14 distinct variants in 11 genes. Only *ATRX* was found mutated in two patients. In addition, of 14 variants, except for the variant in *MECP2*, all the remaining 13 variants are novel, expanding the mutations spectrum, especially for genes *EFNB1, NAA10, LAMA1* and *NFIX*, which are identified recently and only a few of mutations have been reported so far. It is the first time to report mutations in *EFNB1, NAA10, DHCR7, LAMA1* and *NFIX* in Chinese ID/DD patients and first report about mutation in *NAA10* and *LAMA1* in ID/DD patients of Asian ancestry.

The high rate of de novo variants (57.14%) is remarkable, which account for 72.72% (8/11) of patients, not only among autosomal dominant conditions but also in X-linked conditions. The critical role of de novo variants in ID/DD has been reported previously [7, 28–30]. However, it is important to note that many factors have to be evaluated when using the de novo evidence criteria, PS2 and PM6, to judge the pathogenicity of variants [23–25]. For instance, in this study, the testing strategy was gene panel followed by parental testing of variant and being without confirmation of paternity and maternity. Therefore, PS2 could not be used here. Whether PM6 can be applied depends on the consistency and specificity of the phenotype, the number of de novo observations and the inheritance.

Interpreting the variants correctly and achieving a robust genetic diagnosis is still a considerable challenge. Beside the origin of the variant like de novo discussed above, many other factors such as variant allele frequencie, inheritance model and patient’s phenotype should also be evaluated carefully to determine if the variants impair gene function and underlie the phenotype. In this study, after comprehensive analysis, 9 of 112 patients obtained definite diagnosis with a diagnostic rate of 8.03% eventually. Diagnostic rate of targeted NGS is associated with multiple factors including 1) features of study subjects such as gender, severity of ID/DD, with positive family history/complications or not, 2) genes included in the panel, 3) test strategy like gene panel followed by parental testing of variant or NGS for trios/larger family group, 4) data analysis pipeline and definition of the “diagnosis”. Previous studies using targeted NGS in ID/DD led to a conclusive diagnostic rate of 11%~ 32% [6–9]. The yield of this study is lower than that reported. It may be due to that, in this study, 1) subjects were not limited in “syndromic ID/DD” or “moderated to severe ID/DD,” 2) the number of studied genes is relatively small, 3) only proband underwent NGS. For one patient with unexplained ID/DD, more genes and more family members sequenced help increase the chance of diagnosis. However, it also means higher costs. Clinicians and geneticists should weigh the cost effectiveness.

Of 9 patients (Patient 1–6,8–10) with confirmed diagnoses, eight were genetically diagnosed with craniofrontonasal dysplasia (MIM# 304110) (Patient 1), Rett syndrome (MIM# 312750) (Patient 2), X-linked mental retardation-hypotonic facies syndrome (MIM# 309580) (Patient 3, 4), Smith-Lemli-Opitz syndrome (MIM# 270400) (Patient 6), Sotos syndrome (MIM# 614753) (Patient 8), Angelman syndrome (MIM# 105830) (Patient 9) and Coffin-Siris syndrome 1 (CSS1, MIM# 135900) (Patient 10), respectively, whose phenotypes were concordant with the corresponding disorders. Condition of the remaining Patient 5 was relatively complicated.

Patient 5 harbored two promising variants in two different genes *NAA10* and *ANKRD11*. The variant c.248G > A; p.(Arg83His) in *NAA10* is absent in control population, located in the N-acetyltransferase domain and predicted to be deleterious in silico. A different missense change at the same 83 amino acid residue p.(Arg83Cys) is classified as “pathogenic” by three submitters in ClinVar database [31]. *NAA10* is located in Xq28 and mono-allelic mutation in *NAA10* can cause non-syndromic ID/DD in both males and females [32, 33]. The main clinical features of Patient 5 including DD, hyperactivity, electrocardiographic T-wave abnormality and delayed bone age, were all previously reported in other patients with *NAA10* mutations [32, 33]. Moreover, his mother, in heterozygous state for the variant in *NAA10,* also demonstrated slight intellectual defect. The variant in *NAA10* is classified as “likely pathogenic” based on evidence criteria PM1, PM2, PM5, PM6, PP3 and PP4. The variant c.884G > A; p.(Ser295Asn) in *ANKRD11* arose de novo in the patient. Heterozygous mutations in *ANKRD11* lead to KBG syndrome, which is characterized by macrodontia of the upper central incisors, distinctive craniofacial findings, short stature, skeletal anomalies and neurologic symptoms including ID/DD and seizures [34]. Phenotypic heterogenicity is significant in ANKRD11-related KBG syndrome and none of the features mentioned above is a prerequisite for diagnosis. The variant in *ANKRD11* in Patient 5 is classified as “uncertain significance” based on evidence criteria PM2 (absent in population database), while the de novo evidence criteria PS2 or PM6 cannot be used due to lack of confirmation of the paternity and maternity and unspecific phenotype with high genetic heterogeneity. It is unclear if the variant in *ANKRD11* contributes to the phenotype of Patient 5.

It is worth noting that the variant in *ARID1B* in Patient 10 is a missense variant. Mono-allelic mutations in *ARID1B* lead to CSS1. The common features of CSS1 are ID/DD, speech delay, coarse facies, hypertrichosis, small fifth finger or toenails, feeding difficulties [35]. Agenesis of the corpus callosum, seizures, myopia, growth delay, abnormal dentition and single transverse palmar crease are also recorded in some patients [35]. Of 98 reported pathogenic or likely pathogenic mutations in *ARID1B*, only 3 (3/98, 3.06%) are missense variants and all the remaining are truncating variants (91/98, 92.85%) or splice site variants (4/98, 4.08%) [36]. Phenotypes of Patient 10 including DD, classical coarse face, delayed myelination and agenesis of the splenium are highly concordant with CSS1. Therefore, although missense mutations are rare in *ARID1B*, combining the variant in silico features (absent in control population, conserved, predicted to be damaging) and consistent inheritance pattern (arising de novo, auto-dominant), we propose that the missense variant in *ARID1B* is the genetic cause of the patient. This finding further confirms the point that missense variants in *ARID1B*, which may lead to gain of function or dominant negative effect, can also result in disease.

Patient 7 did not get definite diagnosis after comprehensive analysis. The patient presented with two compound heterozygous variants c.1711_1712del; p.(Ala571ProfsTer8) and c.2755G > C; p.(Gly919Arg) in *LAMA1*. Bi-allelic mutation in *LAMA1* causes a cerebellar dysplasia syndrome named as Poretti-Boltshauser syndrome (PBS) (MIM# 615960) [37]. Recently, Whiffin et al. have presented a statistical framework for calculating the maximum credible population allele frequency (AF) of pathogenic variant based on disease inheritance mode, prevalence, genetic and allelic heterogeneity, penetrance and sampling variance [38]. PBS is an autosomal recessive disorder with a prevalence estimated to be less than 1/1000,000 [39]. Characterized by cerebellar dysplasia with cysts with an enlarged, elongated and square-like shaped fourth ventricle on neuroimaging, phenotype of PBS is highly specific [40, 41]. And, at the moment, bi-allelic variants in *LAMA1* is the only cause of PBS, revealing its unobvious genetic heterogeneity. Up to date, 20 PBS families with bi-allelic variants in *LAMA1* have been published [37, 40]. The two variants in *LAMA1* in Patient 7 have not been reported before. Therefore, maximum allelic contribution (or allelic heterogeneity) is 1/(21 × 2). There is no published evidence that bi-allelic loss-of-function variants in *LAMA1* do not cause PBS. There is only one individual with a homozygous splicing variant in *LAMA1* in gnomAD. Despite of this and considering the huge number of controls in this database, we consider full penetrance. According to Whiffin’s calculator [42], the maximum credible population AF of pathogenic variant in *LAMA1* is 3e-5 (setting the inheritance = biallelic, prevalence = 1/1000,000, genetic heterogeneity = 1, allelic heterogeneity = 0.03, penetrance = 1). The frameshift variant c.1711_1712del; p.(Ala571ProfsTer8) is absent from population databases and is predicted to lead to truncation of the protein at 578th amino acid residue. Several variants in *LAMA1* leading to longer truncated protein have been reported to be pathogenic. Therefore, the variant c.1711_1712del; p.(Ala571ProfsTer8) is supposed to be pathogenic (PM2, PVS1). The frequency of missense variant c.2755G > C; p.(Gly919Arg) in East Asian control population is 1.85e-3, although never in the homozygous state (gnomAD). 1.85e-3 is much higher than the 3e-5, suggesting that the missense variant may be benign (BS1). However, the missense variant is predicted to be damaging by multiple prediction software and is in trans with the pathogenic truncated variant, which are supporting (PP1) and moderate evidence (PM3) for its pathogenicity, respectively. Based on evidence above, the missense variant is classified as “uncertain significance.” Although presenting with ID, the patient did not show other typical features of PBS like ataxia or ocular anomalies [37, 40]. It is also unclear if the patient has cerebellar dysplasia or not, the essential feature of PBS. Her epilepsy and sinus block have also not been reported in patients with PBS before. Therefore, the patient’s diagnosis remained uncertain.

Besides, one de novo variant in candidate gene *PTPRD* was detected. *PTPRD* is a receptor-type protein-tyrosine phosphatase and highly expressed in the human brain (HPA RNA-seq normal tissues) [43], especially in neurons and oligodendrocytes [44]. Ptprd-deficient mice exhibited learning impairment, and Ptprd is an important regulator of synaptic plasticity [45]. It has been proved that PTPRD interacts with IL1RAPL1, mutations in which lead to non-syndromic ID (MIM# 300143). In silico, residual variation intolerance score (RVIS) [46] and pLI score [13] of *PTPRD* is “-3.08(4.8%)” and “1”, respectively, which suggests that the gene is intolerant to functional genetic variant and loss of function variant, respectively. Although no intragenic mutation in *PTPRD* has been reported in ID/DD patients yet, Choucair N et al. [47] found a homozygous *PTPRD* gene microdeletion in one patient with trigonocephaly, hearing loss, and ID. Recently, Gao K et al. [48] found that *PTPRD* combining with *BTD, GALNT10, NMUR2, AUTS2* and *DLG2* constructs a small epilepsy and ID/DD related gene network. All above information suggests that *PTPRD* is a promising candidate gene of ID/DD. Our case provides more evidence for the association between *PTPRD* and ID/DD.

## Conclusions

Here, through targeted NGS of 454 genes and comprehensive analysis, we help 8.0% of patients get genetic diagnoses. It confirms the effectiveness of the test strategy. The study emphasizes the high genetic heterogenicity of Chinese ID/DD patients and the important role of de novo variants. Its findings further ascertain related genes as causative genes of ID/DD, delineate the corresponding phenotypes and expand the mutation spectrum. Identification of the variant in *PTPRD* provides more evidence to support its involvement in ID/DD.

## Additional file

Additional file 1: Gene List. List of 454 genes related to ID/DD. (DOCX 13 kb)

## Abbreviations

1000G: 1000 Genomes Project
CMA: chromosomal microarray analysis
CNVs: copy number variants
CSS1: Coffin-Siris syndrome 1
ESP: Exome Sequencing Project
ExAC: Exome Aggregation Consortium
FISH: fluorescence in situ hybridization
gnomAD: Genome Aggregation Database
ID/DD: intellectual disability/developmental delay
MLPA: multiplex ligation-dependent probe amplification
NGS: next generation sequencing
PBS: Poretti-Boltshauser syndrome
RVIS: residual variation intolerance score
WES: whole exome sequencing
WGS: whole genome sequencing
WISC: Wechsler Intelligence Scale for Children

## References

[1] Moeschler JB, Shevell M (2014). Committee on G. Comprehensive evaluation of the child with intellectual disability or global developmental delays. Pediatrics..

[2] Vissers LE, Gilissen C, Veltman JA (2016). Genetic studies in intellectual disability and related disorders. Nat Rev Genet..

[3] Wright CF, FitzPatrick DR, Firth HV (2018). Paediatric genomics: diagnosing rare disease in children. Nat Rev Genet.

[4] Mefford HC, Batshaw ML, Hoffman EP (2012). Genomics, intellectual disability, and autism. N Engl J Med.

[5] Sun Y, Ruivenkamp CA, Hoffer MJ, Vrijenhoek T, Kriek M, van Asperen CJ (2015). Next-generation diagnostics: gene panel, exome, or whole genome?. Hum Mutat.

[6] Martinez F, Caro-Llopis A, Rosello M, Oltra S, Mayo S, Monfort S (2017). High diagnostic yield of syndromic intellectual disability by targeted next-generation sequencing. J Med Genet.

[7] Redin C, Gerard B, Lauer J, Herenger Y, Muller J, Quartier A (2014). Efficient strategy for the molecular diagnosis of intellectual disability using targeted high-throughput sequencing. J Med Genet.

[8] Grozeva D, Carss K, Spasic-Boskovic O, Tejada MI, Gecz J, Shaw M (2015). Targeted next-generation sequencing analysis of 1,000 individuals with intellectual disability. Hum Mutat.

[9] Han JY, Jang JH, Park J, Lee IG (2018). Targeted next-generation sequencing of Korean patients with developmental delay and/or intellectual disability. Front Pediatr.

[10] McLaren W, Gil L, Hunt SE, Riat HS, Ritchie GR, Thormann A (2016). The Ensembl variant effect predictor. Genome Biol.

[11] Auer PL, Johnsen JM, Johnson AD, Logsdon BA, Lange LA, Nalls MA (2012). Imputation of exome sequence variants into population-based samples and blood-cell-trait-associated loci in African Americans: NHLBI GO exome sequencing project. Am J Hum Genet.

[12] Siva N (2008). 1000 Genomes project. Nat Biotechnol.

[13] Lek M, Karczewski KJ, Minikel EV, Samocha KE, Banks E, Fennell T (2016). Analysis of protein-coding genetic variation in 60,706 humans. Nature..

[14] Ng PC, Henikoff S (2003). SIFT: predicting amino acid changes that affect protein function. Nucleic Acids Res.

[15] Adzhubei IA, Schmidt S, Peshkin L, Ramensky VE, Gerasimova A, Bork P (2010). A method and server for predicting damaging missense mutations. Nat Methods.

[16] Schwarz JM, Cooper DN, Schuelke M, Seelow D (2014). MutationTaster2: mutation prediction for the deep-sequencing age. Nat Methods.

[17] Kircher M, Witten DM, Jain P, O'Roak BJ, Cooper GM, Shendure J (2014). A general framework for estimating the relative pathogenicity of human genetic variants. Nat Genet.

[18] Jagadeesh KA, Wenger AM, Berger MJ, Guturu H, Stenson PD, Cooper DN (2016). M-CAP eliminates a majority of variants of uncertain significance in clinical exomes at high sensitivity. Nat Genet.

[19] Gonzalez-Perez A, Lopez-Bigas N (2011). Improving the assessment of the outcome of nonsynonymous SNVs with a consensus deleteriousness score, Condel. Am J Hum Genet.

[20] Choi Y, Chan AP (2015). PROVEAN web server: a tool to predict the functional effect of amino acid substitutions and indels. Bioinformatics..

[21] Desmet FO, Hamroun D, Lalande M, Collod-Beroud G, Claustres M, Beroud C (2009). Human splicing finder: an online bioinformatics tool to predict splicing signals. Nucleic Acids Res.

[22] Splice site prediction. http://www.fruitfly.org/seq_tools/splice.html. Accessed 17 Sept 2018.

[23] Richards S, Aziz N, Bale S, Bick D, Das S, Gastier-Foster J (2015). Standards and guidelines for the interpretation of sequence variants: a joint consensus recommendation of the American College of Medical Genetics and Genomics and the Association for Molecular Pathology. Genet Med.

[24] ACGS (2018). Best practice guidelines for variant classification.

[25] SVI Recommendation for De Novo Criteria (PS2 & PM6) - Version 1.0. https://www.clinicalgenome.org/site/assets/files/3461/recommendation_ps2_and_pm6_acmgamp_critiera_version_1_0.pdf. Accessed 11 Apr 2019.

[26] Buyse IM, Fang P, Hoon KT, Amir RE, Zoghbi HY, Roa BB (2000). Diagnostic testing for Rett syndrome by DHPLC and direct sequencing analysis of the MECP2 gene: identification of several novel mutations and polymorphisms. Am J Hum Genet.

[27] Cooper GM, Stone EA, Asimenos G, Program NCS, Green ED, Batzoglou S (2005). Distribution and intensity of constraint in mammalian genomic sequence. Genome Res.

[28] de Ligt J, Willemsen MH, van Bon BW, Kleefstra T, Yntema HG, Kroes T (2012). Diagnostic exome sequencing in persons with severe intellectual disability. N Engl J Med.

[29] Hamdan FF, Srour M, Capo-Chichi JM, Daoud H, Nassif C, Patry L (2014). De novo mutations in moderate or severe intellectual disability. PLoS Genet.

[30] Rauch A, Wieczorek D, Graf E, Wieland T, Endele S, Schwarzmayr T (2012). Range of genetic mutations associated with severe non-syndromic sporadic intellectual disability: an exome sequencing study. Lancet..

[31] Landrum MJ, Lee JM, Riley GR, Jang W, Rubinstein WS, Church DM (2014). ClinVar: public archive of relationships among sequence variation and human phenotype. Nucleic Acids Res.

[32] Popp B, Stove SI, Endele S, Myklebust LM, Hoyer J, Sticht H (2015). De novo missense mutations in the NAA10 gene cause severe non-syndromic developmental delay in males and females. Eur J Hum Genet.

[33] Wu Y, Lyon GJ (2018). NAA10-related syndrome. Exp Mol Med.

[34] Sirmaci A, Spiliopoulos M, Brancati F, Powell E, Duman D, Abrams A (2011). Mutations in ANKRD11 cause KBG syndrome, characterized by intellectual disability, skeletal malformations, and macrodontia. Am J Hum Genet.

[35] Santen GW, Clayton-Smith J (2014). Consortium ABC. The ARID1B phenotype: what we have learned so far. Am J Med Genet C Semin Med Genet.

[36] Bogershausen N, Wollnik B (2018). Mutational landscapes and phenotypic Spectrum of SWI/SNF-related intellectual disability disorders. Front Mol Neurosci.

[37] Aldinger KA, Mosca SJ, Tetreault M, Dempsey JC, Ishak GE, Hartley T (2014). Mutations in LAMA1 cause cerebellar dysplasia and cysts with and without retinal dystrophy. Am J Hum Genet.

[38] Whiffin N, Roberts AM, Minikel E, Zappala Z, Walsh R, O'Donnell-Luria AH (2019). Using high-resolution variant frequencies empowers clinical genome interpretation and enables investigation of genetic architecture. Am J Hum Genet.

[39] Orphanet. https://www.orpha.net/consor/cgi-bin/index.php. Accessed 1 March 2019.

[40] Micalizzi A, Poretti A, Romani M, Ginevrino M, Mazza T, Aiello C (2016). Clinical, neuroradiological and molecular characterization of cerebellar dysplasia with cysts (Poretti-Boltshauser syndrome). Eur J Hum Genet.

[41] Wente S, Schröder S, Buckard J, Büttel HM, von Deimling F, Diener W (2016). Nosological delineation of congenital ocular motor apraxia type Cogan: an observational study. Orphanet J Rare Dis.

[42] Frequency Filter. http://cardiodb.org/allelefrequencyapp/. Accessed 1 March 2019.

[43] Fagerberg L, Hallstrom BM, Oksvold P, Kampf C, Djureinovic D, Odeberg J (2014). Analysis of the human tissue-specific expression by genome-wide integration of transcriptomics and antibody-based proteomics. Mol Cell Proteomics.

[44] Zhang Y, Sloan SA, Clarke LE, Caneda C, Plaza CA, Blumenthal PD (2016). Purification and characterization of progenitor and mature human astrocytes reveals transcriptional and functional differences with mouse. Neuron..

[45] Uetani N, Kato K, Ogura H, Mizuno K, Kawano K, Mikoshiba K (2000). Impaired learning with enhanced hippocampal long-term potentiation in PTPdelta-deficient mice. EMBO J.

[46] Petrovski S, Gussow AB, Wang Q, Halvorsen M, Han Y, Weir WH (2015). The intolerance of regulatory sequence to genetic variation predicts gene dosage sensitivity. PLoS Genet.

[47] Choucair N, Mignon-Ravix C, Cacciagli P, Abou Ghoch J, Fawaz A, Megarbane A (2015). Evidence that homozygous PTPRD gene microdeletion causes trigonocephaly, hearing loss, and intellectual disability. Mol Cytogenet.

[48] Gao K, Zhang Y, Zhang L, Kong W, Xie H, Wang J (2018). Large De novo microdeletion in epilepsy with intellectual and developmental disabilities, with a systems biology analysis. Adv Neurobiol.

