# Supplementary material for: Targeted next generation sequencing in 112 Chinese patients with intellectual disability/developmental delay: novel mutations and candidate gene
Source: BMC Med Genet. 2019 May 14;20:80. doi: 10.1186/s12881-019-0794-y (PMC6518638; doi:10.1186/s12881-019-0794-y)
Supplement: Supplementary file 1 — Gene List. List of 454 genes related to ID/DD. (DOCX 13 kb) [file 12881_2019_794_MOESM1_ESM.docx]

**Gene List. List of 454 genes related to ID/DD.**

*ABHD5, ACADS, ACBD6, ACSL4, ACTG1, ADAT3, ADRA2B, AFF2, AFF3, AGA, AGPS, AHI1, ALDH18A1, ALDH3A2, ALG6, ALG9, ALMS1, ALS2, ALX4, AMMECR1, ANKRD11, AP1S2, AP4B1, AP4M1, AP4S1, ARFGEF2, ARHGAP26, ARHGEF6, ARHGEF7, ARHGEF9, ARID1B, ARIH1, ARSE, ASCC3, ASH1L, ASL, ATP8A2, ATR, ATRX, AVPR2, B3GALTL, BBS1, BBS10, BBS12, BBS2, BBS4, BBS5, BBS7, BBS9, BCKDHA, BCKDHB, BCL11A, BCOR, BRAF, BRWD3, BUB1B, C11ORF46, C12ORF57, CA2, CA8, CAMK2G, CAMK4, CAMTA1, CAPRIN1, CARS, CASP2, CBL, CC2D1A, CC2D2A, CCBE1, CCDC22, CDH15, CDK5R1, CDK5RAP2, CDKL3, CDKL5, CDKN1B, CENPJ, CEP152, CEP290, CGGBP1, CHD6, CHKB, CHL1, CHM, CHSY1, CIC, CNKSR1, CNTN4, COL4A3BP, COL4A5, COQ5, COX10, CPEB1, CRBN, CREBBP, CTCF, CTNNB1, CTNND2, CTNS, CTSA, CUX2, CYFIP1, CYFIP2, DAG1, DAXX, DBT, DHCR7, DIP2B, DISC1, DLD, DLG1, DLG3, DLG4, DMRT1, DNAJC6, DOCK3, DOCK8, DOPEY2, DPYD, DYM, DYNC1H1, EFNB1, EFTUD2, EHMT2, EIF2C1, EIF2S3, ELK1, ELP2, ENTPD1, EP300, EPB41L1, ERCC2, ERCC3, ERCC5, ERCC6, ERCC8, ERLIN2, ESCO2, EXOSC3, EXT2, EYA4, EZH2, FASN, FBN1, FBN2, FGD1, FGF13, FGFR2, FGFR3, FH, FKRP, FMR1, FOXC1, FOXG1, FOXP1, FRY, FTCD, FTSJ1, FXR1, FXR2, GABARAP, GALE, GALT, GATAD2B, GDI1, GFER, GIT1, GK, GLI3, GMPPB, GNAS, GNE, GNPAT, GON4L, GPD2, GRIA1, GRIP1, GRM7, GTF2I, GTF2IRD1, HDAC8, HELLS, HERC2, HIST3H3, HIVEP2, HNRNPK, HNRNPU, HPRT1, HRAS, HSD17B4, HSPD1, HUWE1, IDUA, IGBP1, IGF1, IL1RAPL1, IQSEC2, ISPD, ITCH, KANK1, KANSL1, KAT6B, KCNC3, KCTD11, KDM5A, KDM5B, KDM6A, KDM6B, KIAA1279, KIAA2022, KIF11, KIF1A, KIF5C, KIF7, KIRREL3, KRAS, L2HGDH, LAMA1, LAMP2, LARGE, LARP7, LIG4, LIM2, LIMK1, LINS, LMNA, LRP1, LRP2, LRP5, LRPPRC, MAGED2, MAGT1, MAN1B1, MAN2B1, MAOA, MAP1B, MAP2K1, MAP2K2, MAX, MBD4, MCPH1, MECP2, MED12, MED13L, MED23, MID1, MKKS, MKRN3, MKS1, MLL, MLL2, MLYCD, MPI, MSMO1, MTF1, MTRR, MYCN, MYO5A, MYT1L, NAA10, NAGS, NDST1, NEU1, NF1, NFIX, NHS, NIN, NIPBL, NKX2-1, NLGN1, NLGN3, NLGN4X, NPAS3, NPAS4, NRF1, NSD1, NSUN2, NUFIP2, NXF1, NXF2, NXF3, NXF5, OCRL, OFD1, OMG, OPA3, OPLAH, P2RY8, PAK3, PAPSS2, PAX6, PCNT, PECR, PEPD, PEX7, PGAP2, PGK1, PHACTR1, PHF21A, PHF8, PHIP, PIGT, PITX3, PJA1, PLP2, PMM2, PNP, POLR3B, POMGNT1, POMT1, POMT2, POU1F1, POU3F4, PPOX, PPP2R5D, PQBP1, PRKRA, PRMT1, PRMT10, PROX2, PRPS1, PRSS12, PSMA7, PTCHD1, PTER, PTPRD, PYCR1, RAB3A, RAB3GAP1, RAB3GAP2, RABL6, RAI1, RALGDS, RAPGEF1, RB1, RBMX, RCAN1, REEP1, REST, RGS7, RPGRIP1L, RPL10, RPS19, RPS6KA6, RTN4R, SATB2, SCO1, SETBP1, SETD5, SGCA, SHANK1, SHANK2, SHH, SHROOM4, SIL1, SIM2, SIN3A, SIRT1, SLC12A3, SLC16A2, SLC25A15, SLC26A4, SLC31A1, SLC4A4, SLC9A6, SMAD3, SMAD4, SMARCA1, SMARCB1, SMC1A, SMC3, SMPD1, SMS, SNRPN, SOX3, SOX8, SPG11, SPG20, SRD5A3, SRGAP3, STAG1, STAU1, STRA6, STS, STX1A, SYNCRIP, TAF2, TANC2, TAT, TBR1, TBX3, TCN2, TCOF1, TECR, TH, THOC6, TIMM8A, TMCO1, TMEM135, TNPO2, TRAPPC11, TRAPPC2, TRAPPC9, TRIO, TRPC5, TRPS1, TSHZ1, TSPAN7, TTI2, TUBB3, TUSC3, UBE3A, UBE3B, UBR1, UBR7, UMPS, UPF3B, UROC1, USF2, VCX, VCX2, VCX3A, VCX3B, VPS13B, VPS37A, WAC, WDR45L, WDR5, WDR62, WDR81, WHSC1, WT1, XIST, YY1, ZBTB16, ZBTB18, ZBTB40, ZC3H14, ZC4H2, ZCCHC12, ZCCHC8, ZDHHC15, ZDHHC9, ZFYVE26, ZMYM3, ZNF407, ZNF41, ZNF526, ZNF592, ZNF630, ZNF674, ZNF711, ZNF81.*
